# Supplementary material for: ZNF334 truncation mutation drives cold-induced autoinflammation
Source: EMBO Mol Med. 2025 Oct 30;17(12):3440–71. doi: 10.1038/s44321-025-00328-x (PMC12686423; doi:10.1038/s44321-025-00328-x)
Supplement: Supplementary file 1 — Appendix [file 44321_2025_328_MOESM1_ESM.pdf]

APPENDIX

ZNF334 truncation mutation drives cold-induced autoinflammation

Table of contents:

Appendix	Description	Page
Appendix Figure S1	Relative mRNA expression levels of *NFKB1* and *STAT3* in C10 and SR2 clones of THP-1 monocytes	2
Appendix Figure S2	Analysis of the RNA-seq data of classical monocytes derived from patients with polyarticular juvenile idiopathic arthritis	3
Appendix Table S1	Genetic variants identified by whole exome sequencing of PBMCs derived from our patient	4
Appendix Table S2	Allele frequencies of ZNF334 truncation mutations in gnomAD v.4.1.0	5
Appendix Table S3	Sequencing and PCR primer sequences used in this study	6
Appendix Table S4	Oligo mouse/rat anti-human antibodies	7
Appendix Table S5	Primers for amplicon-based deep sequencing	8-11
Appendix Table S6	Exact p values for figures 2, 4, 5, 6, 8, EV3	12

Appendix Fig. S1

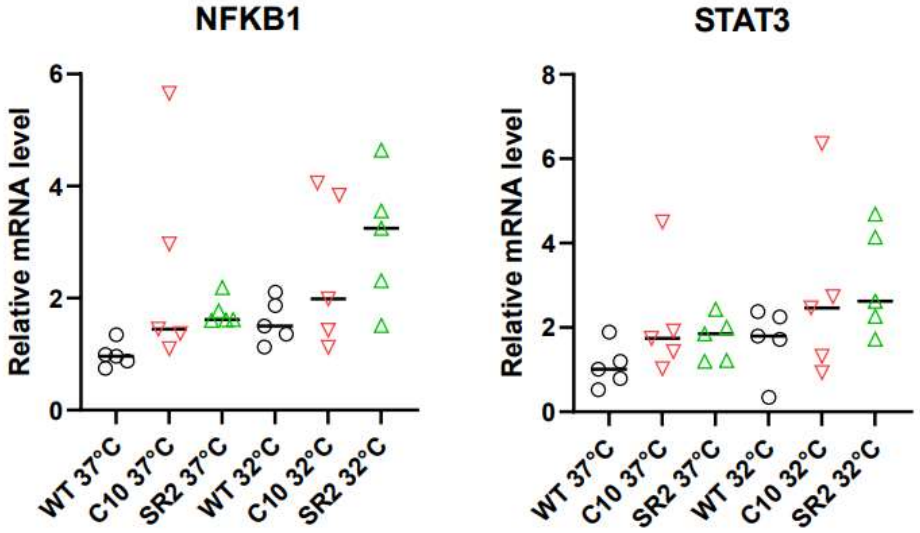

Appendix Fig. S1. Relative mRNA expression levels of *NFKB1* and *STAT3* in ZNF334 wild-type (◯, n = 5), ZNF334+/− (▽, clone C10, the clone used in all experiments of this study, n = 5), and ZNF334+/− (△, clone SR2, n = 5) THP-1 monocytes at 37°C and at 6 h of cold stimulation at 32°C. Relative expression was calculated as the fold change compared with the mean baseline (37°C) expression level in ZNF334 wild-type THP-1 cells. Lines represent median values.

Appendix Fig. S2

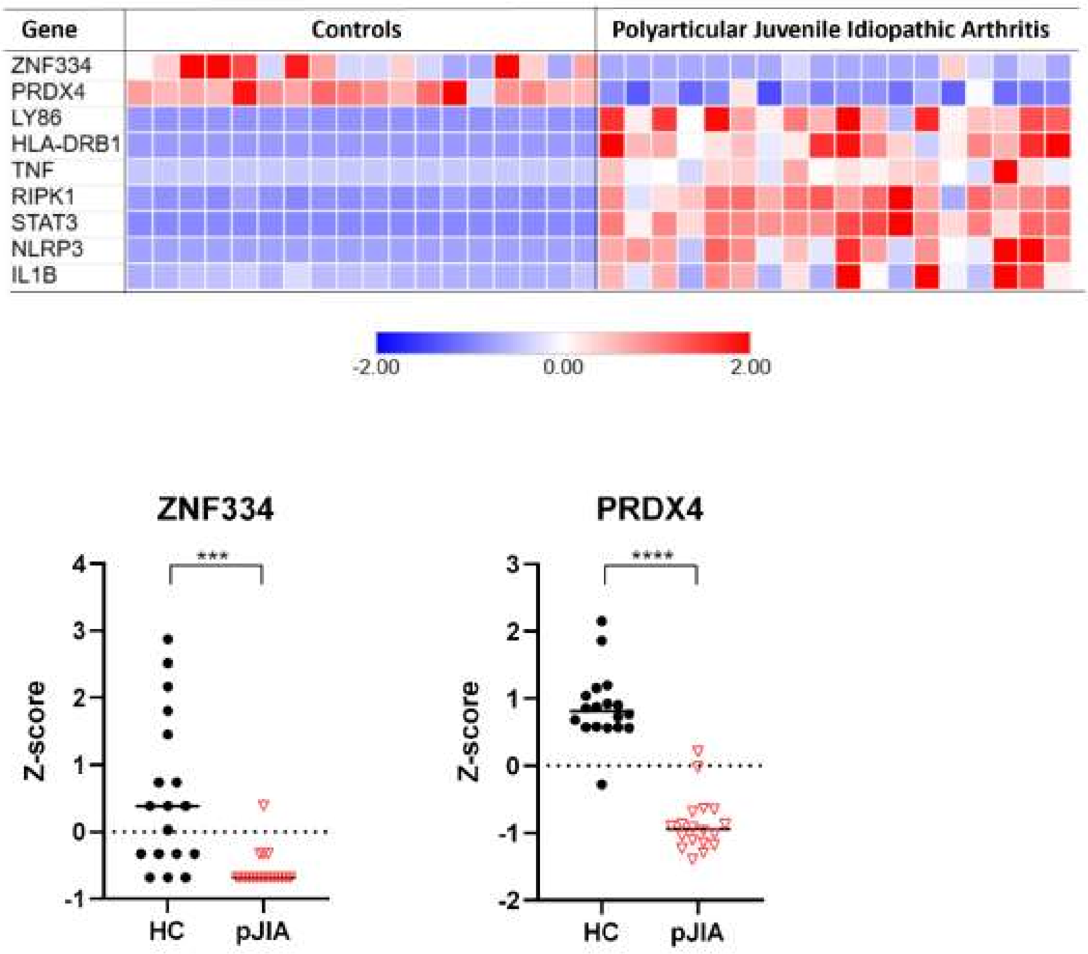

Appendix Fig. S2. Analysis of the RNA-seq data (E-MTAB-14035) of classical monocytes derived from patients with polyarticular juvenile idiopathic arthritis (pJIA) revealed the downregulated expression of *ZNF334* and the oxidative protein folding enzyme *PRDX4*, along with upregulated expression of genes related to monocyte activation and pro-inflammatory cytokines. Heatmap showing z-scores of normalized counts of each gene in the control group (HC) and in the pJIA group is generated via Morpheus (software.broadinstitue.org).***P<0.001, ****P<0.0001, by t test.

Appendix Table S1. Genetic variants identified by whole exome sequencing of PBMCs derived from our patient (Genome build on GRCh37)

Gene	rs number	Variant allele	Protein level	Consequence	General population frequency (ExAC)	East Asian frequency	Taiwan Biobank frequency	Detected in healthy siblings
ZNF334	rs543974571	delCT	p.Thr399fs*	frameshift variant	0.000255	0.0006014	0.000670241	No
CNR2	rs23875227	G>A	p.Arg131Ter	stop gained	0.000214	0.002711	0.0016756	Yes

*East Asian frequency was obtained from gnomAD v2.1.1 controls East Asian (GRCh37)

**Detection of genetic variants in healthy siblings was done by Sanger sequencing

Appendix Table S2. Allele frequencies of ZNF334 truncation mutations in gnomAD v.4.1.0 (GRCh38)

ZNF334 variant	rs number	Total allele frequency	Validation via Sanger sequencing	Functional study	Reported in ClinVar
p.Glu402LysfsTer4[$]	rs543974571	0.0002044	This study	This study	No
p.Glu402LysfsTer52	—	6.195E-07	NA	NA	No
p.Cys407Ter	—	6.195E-07	NA	NA	No
p.Glu409Ter	rs762555233	0.00007125	NA	NA	No
p.Glu396Ter	—	0.000001239	NA	NA	No
p.Lys347AlafsTer31	rs1555854634	0.00008302	NA	NA	No
p.Arg284Ter[$$]	rs202077322	0.0006086	NA	NA	No

[$]: The ZNF334 p.Thr399fs in our patient was identified by sequence alignment to GRCh37 (the reference gnomeAD version was v.2.1.1), the same variant (location 20-45130776-CCT-C) has been lifted over to GRCh38 location 20-46502137-CCT-C, reported in gnomAD v4.1.0 (GRCh38), with the same protein consequence, but designated as p.Glu402LysfsTer4

[$$]: Significantly different frequencies are observed between the whole exome and whole genome data of ZNF334 p.Arg284Ter mutation in gnomAD v4.1, with a Cochran Mantel Haenszel test P value of $5.11e^{-5}$

Appendix Table S3. Sequencing and PCR primer sequences used in this study

Target	Primer	Sequence (5'-3')
ZNF334	Forward (sequencing primer)	CAATGAGGGCTGATTTCGTA
	Reverse (sequencing primer)	AGCCTTACGAATGCAAGGAA
ZNF334	Forward (PCR primer)	GCACCCACAACCTATAGGAAG
	Reverse (PCR primer)	GGTCCTGGAATGAAACTGGTAT
NFKB1	Forward (PCR primer)	GGAGCACGACAACATCTCATTG
	Reverse (PCR primer)	GGTGTGGTTCCATCGTAGGTA
RIPK1	Forward (PCR primer)	TATCCCAGTGCCTGAGACCAAC
	Reverse (PCR primer)	GTAGGCTCCAATCTGAATGCCAG
RIPK3	Forward (PCR primer)	GCTACGATGTGGCGGTCAAGAT
	Reverse (PCR primer)	TTGGTCCCAGTTCACCTTCTCG
NLRP3	Forward (PCR primer)	CGTGTTCACTGCCTGGTATC
	Reverse (PCR primer)	AGCGGGTGCTTGCCATCTTC
STAT3	Forward (PCR primer)	GCAGTTTCTTCAGAGCAGGTATC
	Reverse (PCR primer)	AGGCACCGGGCCACAATC
TNF	Forward (PCR primer)	GGCCCGACTATCTCGACTTTG
	Reverse (PCR primer)	AGGCGTTTGGGAAGGTTGGAT
IL-6	Forward (PCR primer)	ACTCACCTCTTCAGAACGAATTG
	Reverse (PCR primer)	CCATCTTTGGAAGGTTCAGGTTG
PPIB	Forward (PCR primer)	TGATCTTTGGTCTCTTCGGAAA
	Reverse (PCR primer)	CCTGGATCATGAAGTCCTTGA

Appendix Table S4. Oligo mouse/rat anti-human antibodies

Target	Clone	Oligonucleotide barcode sequence
CD45	HI30	GTGCGAAATGGCGGAATGTTATCTGCGAATGTAGTC
CD3	SK7	AAAGGTAGAGTGTATTGACGTCGGTGTAGGTTGATT
CD4	SK3	TCGGTGTTATGAGTAGGTCGTCGTGCGGTTTGATGT
CD8	RPA-T8	TGATTGGGTACGCGCTTGGCTTATATAGTCGGGTCT
CD25	2A3	AGTTGTATGGGTTAGCCGAGAGTAGTGCGTATGATT
CD19	SJ25C1	TAGTAATGTGTTCGTAGCCGGTAATAATCTTCGTGG
CD56	NCAM16.2	AGAGGTTGAGTCGTAATAATAATCGGAAGGCGTTGG
CD14	MPHIP9	TGGCCCGTGGTAGCGCAATGTGAGATCGTAATAAGT
CD16	3G8	TAAATCTAATCGCGGTAACATAACGGTGGGTAAGGT
CD11b	M1/70	ATCGTTATTCGTTGTAGTTCGCCCGGTTTGAGTAGT
CD11c	B-LY6	ATGCGTTGCGAGAGATATGCGTAGGTTGCTGATTGG

Appendix Table S5. Primers for amplicon-based deep sequencing

Primer name	Orientation	Primer Seq (5'-3')
NLRP3_1F	FORWARD	CAGATGAAGATGGCAAGCAC
NLRP3_1R	REVERSE	CGTGGCTAGATCCACATGGT
NLRP3_2F	FORWARD	GGACTATCCTCCCCAGAAGG
NLRP3_2R	REVERSE	TCGGCTCATCTCTTTTTGCT
NLRP3_3F	FORWARD	TTCTGGGTTTTGACACCTTTTT
NLRP3_3R	REVERSE	CTAGAAGCACCACCCCAGTC
NLRP3_4F	FORWARD	CCAGTGCATTGAAGACAGGA
NLRP3_4R	REVERSE	GGGTCAAACAGCAACTCCAT
NLRP3_5F	FORWARD	GCCAGGAAGATGATGTTGGA
NLRP3_5R	REVERSE	TCTCACGATCTTGTGGATGG
NLRP3_6F	FORWARD	ACCCTCCAGAATCCTCTTCC
NLRP3_6R	REVERSE	TCGTGGTGATGAGCAGAGAG
NLRP3_7F	FORWARD	TCTGCACTGACTGGCAGAAG
NLRP3_7R	REVERSE	CTCGGAGAAACCCAGGATCT
NLRP3_8F	FORWARD	GGCAGCCTTCAGTCTGATTC
NLRP3_8R	REVERSE	CAGCAAACTGGAAAGGAAGAA
NLRP3_9F	FORWARD	AAGGAAGTGGACTGCGAGAA
NLRP3_9R	REVERSE	CCCTTTTCGAATTTGCCATA
NLRP3_10F	FORWARD	TTCCAGGGAGTCGTTTGAAG
NLRP3_10R	REVERSE	CAGCCTGATTTGCTGAGAGA
NLRP3_11F	FORWARD	GGAGAGGACCTCCTACTTGG
NLRP3_11R	REVERSE	ATAGTCCATGGCCCTTTGC
NLRP3_12F	FORWARD	TTGCATTGAGAACTGTCATCG
NLRP3_12R	REVERSE	GAAGCCGAGTTTCCTTACCC
NLRP3_13F	FORWARD	TCGAGGCTGATTTCTTTTCTG
NLRP3_13R	REVERSE	TGGAGCGTTTCACACAACAC
NLRC4_1F	FORWARD	ATCATTTGCTGCGAGAAGGT
NLRC4_1R	REVERSE	CTTGCAGAAACAGATGCAAAA
NLRC4_2F	FORWARD	ACTTGGACGATTTGGCTCAG
NLRC4_2R	REVERSE	CAGGGTCAGCTGCTCCAC
NLRC4_3F	FORWARD	GGAAGGACCAACACCATCAC
NLRC4_3R	REVERSE	GAACTTGGTCAGAGCCTTGC
NLRC4_4F	FORWARD	AAGGCAAGTCCACTCTGCTG
NLRC4_4R	REVERSE	TGTCTGCTTCCTGATTGTGC
NLRC4_5F	FORWARD	CCAGGTGCTTGAGGAATCTC
NLRC4_5R	REVERSE	GAAGTCACTTGCAGCCACAC

NLRC4_6F	FORWARD	TGAATGAGGATGTCCTGCTG
NLRC4_6R	REVERSE	TAACCATTCCCCTTGGTCAC
NLRC4_7F	FORWARD	TGGAATCCACATGGAAGAGG
NLRC4_7R	REVERSE	GCTTGTGGCAGAGCTGAATA
PLCG2_1F	FORWARD	CTGCTAAACGGTGTGCTTTG
PLCG2_1R	REVERSE	GATGATGTGTGCACGTACCG
PLCG2_2F	FORWARD	GAAGGCTGACAGCATCATCA
PLCG2_2R	REVERSE	CTGCCGTCTGGAAATTGAGT
PLCG2_3F	FORWARD	TATGACCCAAACCTGGCATT
PLCG2_3R	REVERSE	GCAGCCAAACCAAAGTACAA
PLCG2_ex19F-1	FORWARD	CACTGACAGCCTGGAGACC
PLCG2_ex19R-1	REVERSE	AGGTGATGGCATAGGAGTCG
PLCG2_ex19F-2	FORWARD	GAGGCAGAGGACATGCTGAT
PLCG2_ex19R-2	REVERSE	ACCTGAAGTTCCCCCTTGTC
PLCG2_ex19F-del-1	FORWARD	ACTGGGCCTGGCTAATTTTT
PLCG2_ex19R-del-1	REVERSE	CATGCCTGTAATCCCAGCTA
PLCG2_ex19F-del-2	FORWARD	CAGGGTTTCACCGTGTTAGC
PLCG2_ex19R-del-2	REVERSE	TAAGGAAGTCAAACAGCTCAATAG
PLCG2_ex20F-1	FORWARD	AAAAATTGTTTGGCCACCAG
PLCG2_ex20R-1	REVERSE	CGGTAGAGTGAATGCTTCTCG
PLCG2_ex20F-2	FORWARD	CACTTTGTGCTGGGGACCT
PLCG2_ex20R-2	REVERSE	AATCCCACCAAATCACAAGG
PLCG2_ex21F-1	FORWARD	AAGGGCTATTCCAGGAGCAT
PLCG2_ex21R-1	REVERSE	CAGCCTCGTGACTCCAAAAT
PLCG2_ex22F-1	FORWARD	CAAATGGTACCTGGGAACG
PLCG2_ex22R-1	REVERSE	TGGAGACATTGTGGATGAGG
PLCG2_ex22F-2	FORWARD	ACTACAAAGCCAAGCGAAGC
PLCG2_ex22R-2	REVERSE	GACAAAGGGGGTCAGACTTG
PLCG2_ex20-22F-del	FORWARD	CAAGTGATCTGCCTGCCTTG
PLCG2_ex20-22R-del	REVERSE	CCTGCCCATCTCTTGAGCTA
NLRP12_1F	FORWARD	AAGAACTCGAGGCTGTGGAA
NLRP12_1R	REVERSE	AGGTGCTGAGAGCCAACCT
NLRP12_2F	FORWARD	AGCAGCAGCTTCTGGACAC
NLRP12_2R	REVERSE	GTCCAGCATCACCTTGTGTG
NLRP12_3F	FORWARD	GAAGCTCTTCCAAGGCAGATT
NLRP12_3R	REVERSE	AAGCCGTCGATGATGAAAAG
NLRP12_4F	FORWARD	CTCCAGGAGCTCATCCGAGT
NLRP12_4R	REVERSE	GATAGCTCAGGGAGCAGCTT

NLRP12_5F	FORWARD	CCTCTGCTGGGAGGAGAAAC
NLRP12_5R	REVERSE	CCTCAGAGAAGCCCAGGAT
NLRP12_6F	FORWARD	CAATTACGTGAGGGACAACG
NLRP12_6R	REVERSE	TTGGGTTGCATCAGACTCAG
NLRP12_7F	FORWARD	CAGACCAGGACGTGACCAG
NLRP12_7R	REVERSE	ATCCACTGCAACAGGTCCAT
NLRP12_8F	FORWARD	TGTATGGCGCCACCTACAG
NLRP12_8R	REVERSE	AGGTCACGCCAGTGTACTCC
NLRP12_9F	FORWARD	CTGGACGCCTACAGTGAACA
NLRP12_9R	REVERSE	CAGACCAGCCTGCACTCAC
NLRP12_10F	FORWARD	TGGAGCAAAAGAAATGACCA
NLRP12_10R	REVERSE	AGCATCATGCCTGGGAATC
NLRP12_11F	FORWARD	GGGAGACAGGGTGAGACACT
NLRP12_11R	REVERSE	GGTCAGGTAAAGGTCGGTCA
TNFRSF1A_1F	FORWARD	TATTGGACTGGTCCCTCACC
TNFRSF1A_1R	REVERSE	GAAGCAGAGAAAGAAGCAGCA
TNFRSF1A_2F	FORWARD	CTGGCTGTTGTCCCTAGCAT
TNFRSF1A_2R	REVERSE	GGCAGTGTCTGAGGTGGTTT
TNFRSF1A_3F	FORWARD	GGGGCAGGATACGGACTG
TNFRSF1A_3R	REVERSE	CACCAGCCTGCACATAGACA
TNFRSF1A_4F	FORWARD	TCTTCTCCCCTCACCAGAAA
TNFRSF1A_4R	REVERSE	TGCGCTCACAGGAGAGGT
TNFRSF1A_5F	FORWARD	AATGGTAGGGCCTCTGTTCA
TNFRSF1A_5R	REVERSE	GGGCAAGAAGAGGGAGAGG
TNFRSF1A_6F	FORWARD	AGAGGGGACCACGAGAGG
TNFRSF1A_6R	REVERSE	GAATTCCTTCCAGCGCAAC
ZNF334_1F	FORWARD	GCAAAATTGGAGTCAGCACA
ZNF334_1R	REVERSE	GGTTCCCTGACAATGGAGAA
ZNF334_2F	FORWARD	GCATCTTCATTGCACAGCTT
ZNF334_2R	REVERSE	CCTCCACTATCCATGGCTCT
ZNF334_3F	FORWARD	TTGGTCCAAGTTCTGTAATTTCC
ZNF334_3R	REVERSE	CCACCATGGAACTCTCATTTC
ZNF334_4F	FORWARD	TAAAGGCTCACCAATTACCG
ZNF334_4R	REVERSE	GGAACACTATTCATGCCCAGA
ZNF334_5F	FORWARD	TTTGGGAAAACACTTAATCTGG
ZNF334_5R	REVERSE	TTCTCATGCTTCCCAAATCC
ZNF334_6F	FORWARD	TGTTGCAAAGAAAAGCAAAGAA
ZNF334_6R	REVERSE	CGGTTGTTTCAAAATCTGAATG

ZNF334_7F	FORWARD	AGGAAAGCCAGCAATCAAAA
ZNF334_7R	REVERSE	TCCTACATTCATTACATTCATTTGG
ZNF334_8F	FORWARD	GGGGAGACAGACTGAAAGGA
ZNF334_8R	REVERSE	TCTTCGGTGTCGAGTGAGG
ZNF334_9F	FORWARD	GGGGAGAAACCGTATGTTTG
ZNF334_9R	REVERSE	TCTCCCCTCCATGAATTTTCT
ZNF334_10F	FORWARD	GCAGGAAAACCTTCATTGACA
ZNF334_10R	REVERSE	GCTGAAGGCATTTCCACATT
ZNF334_11F	FORWARD	TGAACATTTCAGGTCACACACA
ZNF334_11R	REVERSE	GCAGTAAGGGCTGACTGACA
ZNF334_12F	FORWARD	TCACAGAGGAGAGAAGCCAAA
ZNF334_12R	REVERSE	TGTATGACTTCTTCGATGCACA
ZNF334_13F	FORWARD	TGAGAAAACCTTCTTTTGTCAATC
ZNF334_13R	REVERSE	CCCACATTCATTACATTCATAAGAC
ZNF334_14F	FORWARD	GAAATCAGCCCTCATTGCAC
ZNF334_14R	REVERSE	GCAGTTTGACTTCACAATGGAG
ZNF334_15F	FORWARD	TCAGAGAACACACACAGGAGAGA
ZNF334_15R	REVERSE	TCTCTGATGTACAATGAGGTGTGA
ZNF334_16F	FORWARD	TCTCCCAAACAGGATACAAAATC
ZNF334_16R	REVERSE	TGAGTTCCTCTTTGTAAAGCTGTC
ZNF334_17F	FORWARD	TGTTTGGAAGCCTAGGACTCTT
ZNF334_17R	REVERSE	GCTCTTCCTTCCCCCTTCTA

Appendix Table S6. Exact p values for figures 2, 4, 5, 6, 8, EV3.

**Fig.2A**	TNF	IL1-ß	IL-6	eHSP90								
	0.0004	0.0019	0.0017	0.0014								
**Fig.2F**	S100A4	S100A4	S100A6	S100A6	CD44	CD44	NFKB2	NFKB2	NFKBIA	NFKBIA	BCL3	BCL3
	WT_S2	WT_S3	WT_S2	WT_S3	WT_S2	WT_S3	WT_S2	WT_S3	WT_S2	WT_S3	WT_S2	WT_S3
	3.00E-06	0.73	1.30E-06	0.16	0.012	0.0051	0.011	5.80E-05	8.10E-05	0.00014	0.00035	0.036
**Fig.2G**	ANXA1	ANXA1	IL32	IL32	PIM2	PIM2	NFKB2	NFKB2	NFKBIA	NFKBIA	BCL3	BCL3
	WT_S2	WT_S3	WT_S2	WT_S3	WT_S2	WT_S3	WT_S2	WT_S3	WT_S2	WT_S3	WT_S2	WT_S3
	2.10E-06	0.068	7.00E-04	0.86	4.50E-08	0.00014	2.40E-07	1.60E-04	9.70E-11	0.00024	4.40E-06	0.0019
**Fig.4O**	37◦C	32◦C										
	<0.0001	<0.0001										
**Fig.4P**	37_WT vs +/-	37_WT vs -/-	37_3 samples	32_WT vs +/-	32_WT vs -/-	32_3 samples						
	<0.0001	0.0201	<0.0001	0.0001	0.0033	0.0003						
**Fig.4Q**	37_WT vs +/-	37_WT vs -/-	37_3 samples	32_WT vs +/-	32_WT vs -/-	32_3 samples						
	<0.0001	<0.0001	<0.0001	<0.0001	0.0003	<0.0001						
**Fig.5A**	37_WT vs +/-	37_WT vs -/-	32_WT vs +/-	32_WT vs -/-								
	0.0156	0.0156	0.1094	0.5781								
**Fig.5B**	37◦C	32◦C										
	<0.0001	<0.0001										
**Fig.5C**	37◦C	32◦C										
	0.0686	0.0029										
**Fig.6B**	TNF 37	TNF 32	RIPK1 37	RIPK1 32	RIPK3 37	RIPK3 32	NLRP3 37	NLRP3 32				
	0.0312	0.0312	0.0312	0.4375	0.0312	0.1562	0.0625	0.0625				
**Fig.8A**	TNF_WT	TNF_ZNF334+/-	NLRP3_WT	NLRP3_ZNF334+/-	IL6_WT	IL6_ZNF334+/-						
	(inhibitor - vs +)	(inhibitor - vs +)	(inhibitor - vs +)	(inhibitor - vs +)	(inhibitor - vs +)	(inhibitor - vs +)						
	0.0625	0.5	0.1875	0.3125	0.4375	0.4375						
**Fig.8B**	TNF_WT	TNF_ZNF334+/-	NLRP3_WT	NLRP3_ZNF334+/-	IL6_WT	IL6_ZNF334+/-						
	(inhibitor - vs +)	(inhibitor - vs +)	(inhibitor - vs +)	(inhibitor - vs +)	(inhibitor - vs +)	(inhibitor - vs +)						
	0.0312	0.0312	0.3125	0.0312	0.0938	0.5						
**Fig.8C**	TNF_WT	TNF_ZNF334+/-	NLRP3_WT	NLRP3_ZNF334+/-	IL6_WT	IL6_ZNF334+/-						
	(inhibitor - vs +)	(inhibitor - vs +)	(inhibitor - vs +)	(inhibitor - vs +)	(inhibitor - vs +)	(inhibitor - vs +)						
	0.0312	0.0312	0.3125	0.0312	0.5	0.3125						
**Fig.8D**	TNF_WT	TNF_ZNF334+/-	NLRP3_WT	NLRP3_ZNF334+/-	IL6_WT	IL6_ZNF334+/-						
	(inhibitor - vs +)	(inhibitor - vs +)	(inhibitor - vs +)	(inhibitor - vs +)	(inhibitor - vs +)	(inhibitor - vs +)						
	0.1562	0.1562	0.4062	0.4062	0.4062	0.3125						
**Fig.EV3**	TNF	RIPK3	NFKB1	STAT3								
	0.0002	0.0008	0.0003	0.004								
