# Supplementary material for: ZNF334 truncation mutation drives cold-induced autoinflammation
Source: EMBO Mol Med. 2025 Oct 30;17(12):3440–71. doi: 10.1038/s44321-025-00328-x (PMC12686423; doi:10.1038/s44321-025-00328-x)
Supplement: Supplementary file 3 — Expanded View Figures [file 44321_2025_328_MOESM3_ESM.pdf]

Expanded View Figures

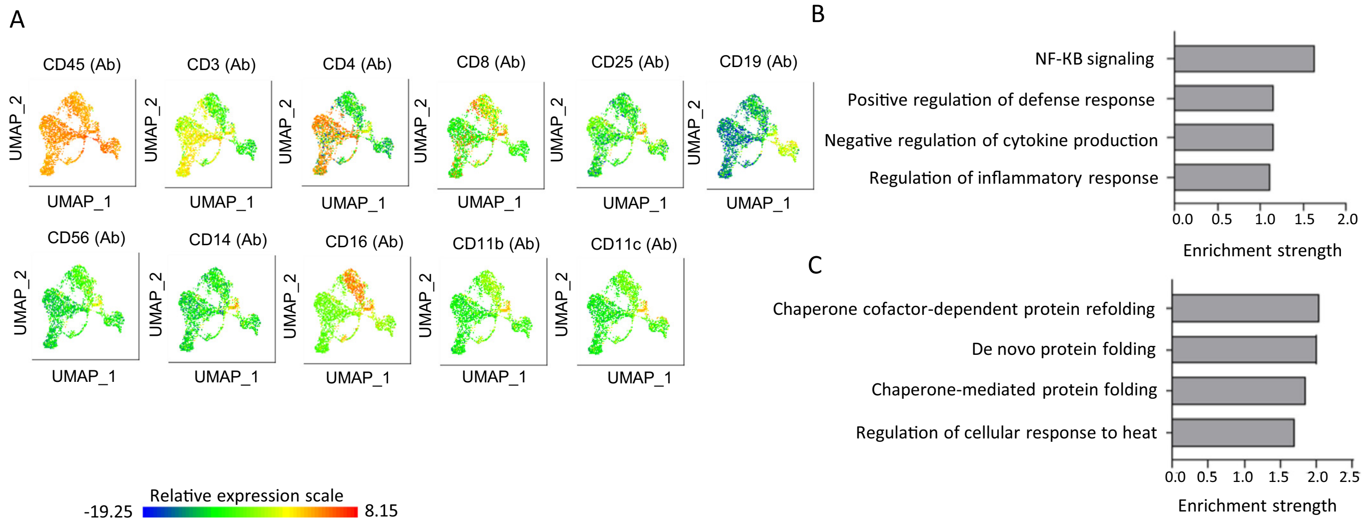

Figure EV1. Single cell analysis of PBMCs.

(**A**) UMAPs of PBMCs annotated by the expression of each oligonucleotide-conjugated antibody targeting a cell surface marker. (**B**) Top four enriched pathways of the top 30 upregulated genes in the cluster annotated as “leukocyte regulating NF-κB signaling.” (**C**) Top four enriched pathways of the top 30 upregulated genes in the cluster annotated as “myeloid cell regulating stress response.”

© The Author(s)

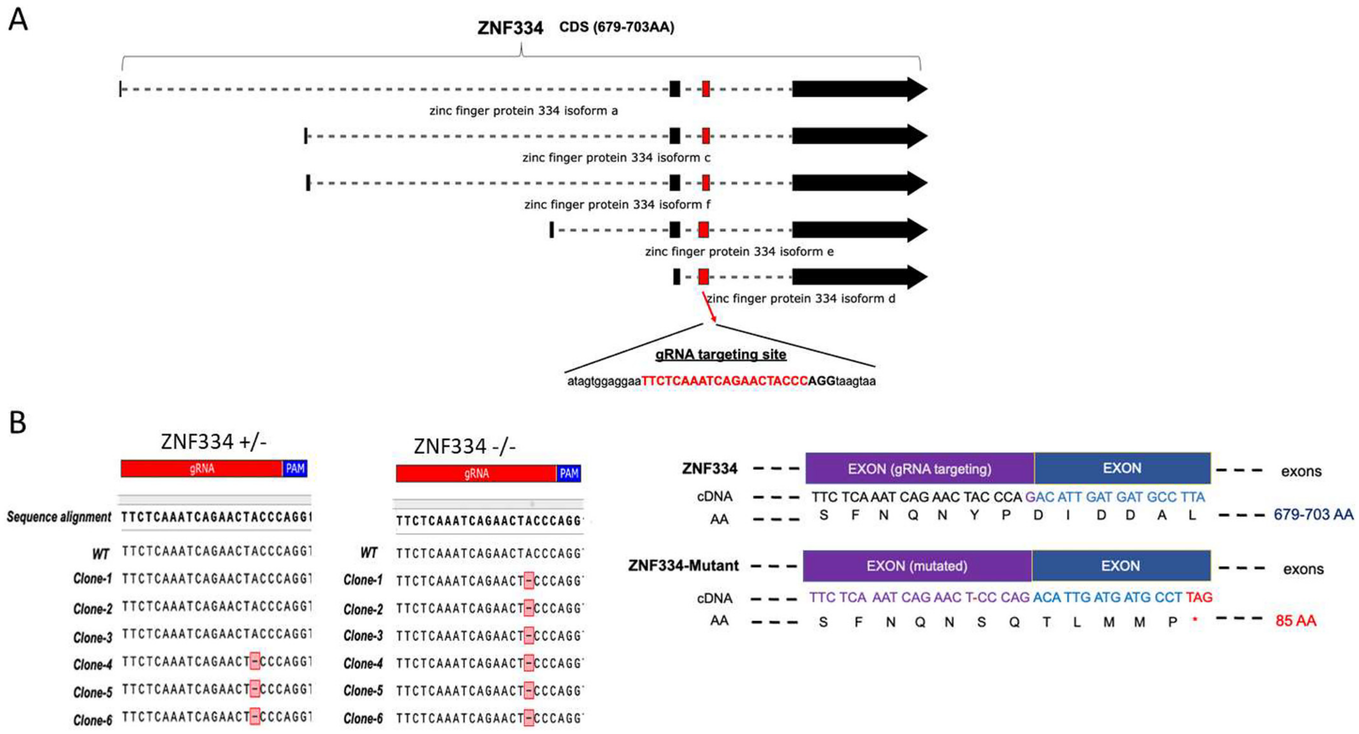

Figure EV2. CRISPR/Cas9-mediated gene knockout of *ZNF334* in the THP-1 cell line.

(**A**) Schematic of the coding transcripts of ZNF344 and the gRNA targeting site in the exon, which is present in all transcripts. (**B**) *ZNF334*-mutant clone has an "A" base deletion in the gRNA targeting exon of ZNF334, which produces a premature stop codon (PTC) on the mutant allele. The mutant allele is predicted to create a short, truncated protein (85 amino acids).

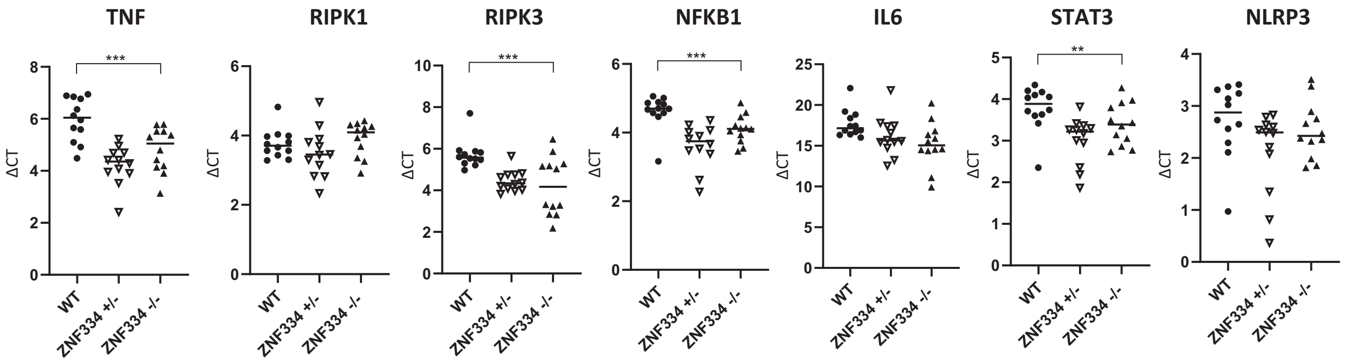

Figure EV3. Basal RNA expression of genes associated with inflammatory/stress response pathways in ZNF334 wild-type, ZNF334$^{+/-}$, and ZNF334$^{-/-}$ THP-1 cells.

Relative RNA expression levels are presented as the ΔCt values of *TNF*, *RIPK1*, *RIPK3*, *NFKB1*, *IL-6*, *STAT3*, and *NLRP3*. THP-1 ZNF334 wild-type, $n = 12$; ZNF334 $+/-$, $n = 12$; ZNF334 $-/-$, $n = 12$. ΔCt = Ct value of the target gene − Ct value of *PPIP* (housekeeping gene). $^{**}P < 0.01$ and $^{***}P < 0.001$: Kruskal–Wallis test. Lines represent medians.

 © The Author(s)

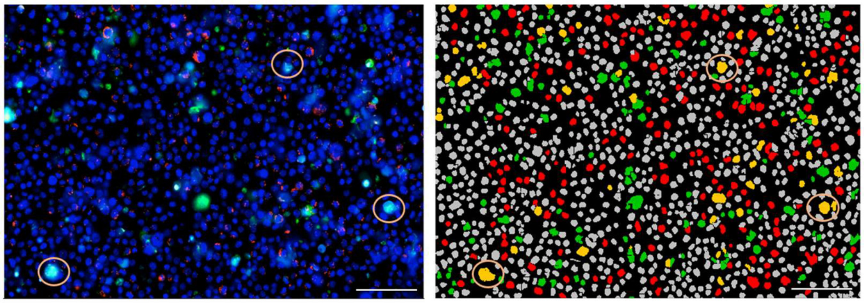

Figure EV4. Representative image of ZNF334$^{+/-}$ THP-1 cells after 4 h of cold stimulation at 32 °C.

Representative necroptotic cells are circled. Left: Annexin V (green) and PI (red). Right: yellow masks indicate necroptotic cells positive for nuclei stain (gray) and Annexin V (green) and PI with an intensity of >10,000 gray levels above the background (red). Images were acquired using the Celldiscoverer7 Micro wide-field fluorescence microscope (ZEISS) and analyzed using the MetaXpress software. Scale bar, 100 μm.

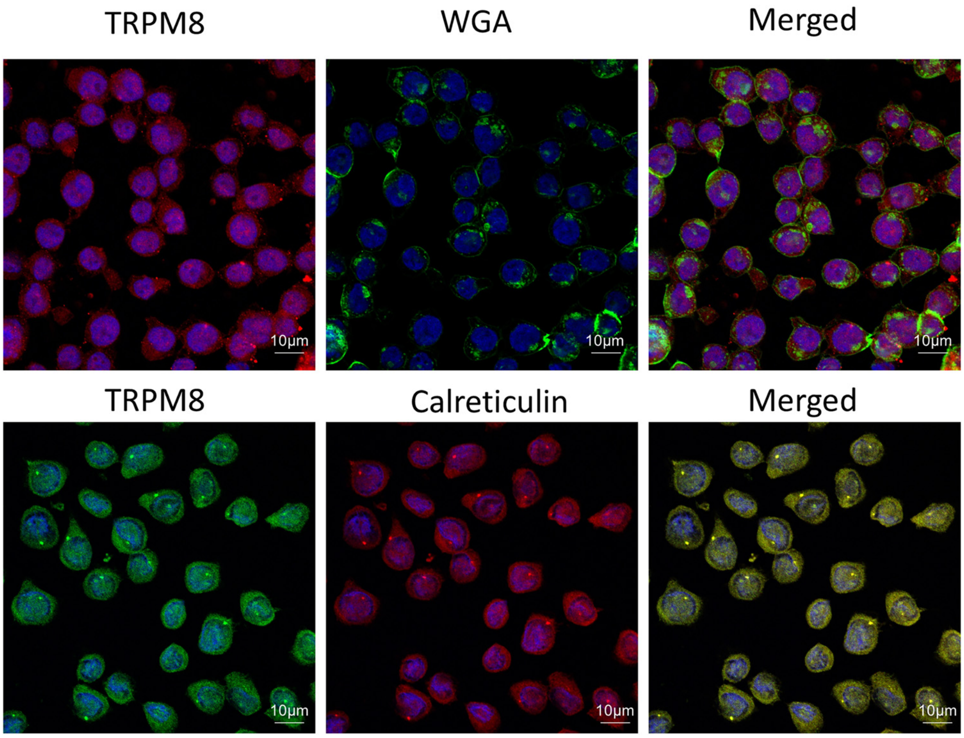

Figure EV5. Colocalization of TRPM8 with WGA and Calreticulin.

THP-1 monocytes (ZNF334 wild-type, at baseline condition) were stained with TRPM8, WGA (plasma membrane stain), calreticulin (ER stain), and DAPI (nuclear stain, blue). Upper graphs: Representative confocal microscopic images showing colocalization of TRPM8 and WGA. Lower graphs: representative confocal microscopic images showing colocalization of TRPM8 and calreticulin.

 © The Author(s)
